# A Multifaceted Computational Approach to Understanding the MERS-CoV Main Protease and Brown Algae Compounds’ Interaction

**DOI:** 10.3390/md21120626

**Published:** 2023-11-30

**Authors:** Hattan S. Gattan, Maha Mahmoud Alawi, Leena H. Bajrai, Thamir A. Alandijany, Isra M. Alsaady, Mai M. El-Daly, Vivek Dhar Dwivedi, Esam I. Azhar

**Affiliations:** 1 Special Infectious Agents Unit-BSL3, King Fahd Medical Research Center, King Abdulaziz University, Jeddah 21362, Saudi Arabia; hsqattan@kau.edu.sa (H.S.G.); malalawi@kau.edu.sa (M.M.A.); lbajrai@kau.edu.sa (L.H.B.); talandijany@kau.edu.sa (T.A.A.); meldaly@kau.edu.sa (M.M.E.-D.); 2 Department of Medical Laboratory Sciences, Faculty of Applied Medical Sciences, King Abdulaziz University, Jeddah 21362, Saudi Arabia; 3 Department of Clinical Microbiology and Immunology, Faculty of Medicine, King Abdulaziz University, Jeddah 21589, Saudi Arabia; 4 Infection Control & Environmental Health Unit, King Abdulaziz University Hospital, King Abdulaziz University, Jeddah 21589, Saudi Arabia; 5 Biochemistry Department, Faculty of Sciences, King Abdulaziz University, Jeddah 21362, Saudi Arabia; 6 Center for Global Health Research, Saveetha Institute of Medical and Technical Sciences, Saveetha Medical College and Hospitals, Saveetha University, Chennai 605102, India; 7 Bioinformatics Research Division, Quanta Calculus, Greater Noida 201310, India

**Keywords:** MERS-CoV, brown algae, main protease, molecular dynamics

## Abstract

Middle East Respiratory Syndrome (MERS) is a viral respiratory disease caused b a special type of coronavirus called MERS-CoV. In the search for effective substances against the MERS-CoV main protease, we looked into compounds from brown algae, known for their medicinal benefits. From a set of 1212 such compounds, our computer-based screening highlighted four—CMNPD27819, CMNPD1843, CMNPD4184, and CMNPD3156. These showed good potential in how they might attach to the MERS-CoV protease, comparable to a known inhibitor. We confirmed these results with multiple computer tests. Studies on the dynamics and steadiness of these compounds with the MERS-CoV protease were performed using molecular dynamics (MD) simulations. Metrics like RMSD and RMSF showed their stability. We also studied how these compounds and the protease interact in detail. An analysis technique, PCA, showed changes in atomic positions over time. Overall, our computer studies suggest brown algae compounds could be valuable in fighting MERS. However, experimental validation is needed to prove their real-world effectiveness.

## 1. Introduction

The Middle East Respiratory Syndrome Coronavirus, known scientifically as MERS-CoV and classified under the Coronaviridae lineage, initially made its presence felt in Saudi Arabia in 2012 [1]. Its rapid dissemination across national boundaries evoked heightened global alarm, especially given its pronounced morbidity and mortality rates. The chief clinical manifestation of MERS-CoV in humans is an acute respiratory ailment [2]. The symptomatology spans from inconspicuous upper respiratory indicators to life-threatening conditions such as grave pneumonia and acute respiratory distress syndrome (ARDS) [3]. Drawing upon data up to September 2021, this pathogen had compromised the health of around 2500 individuals, leading to nearly 858 fatalities, indicating a grave fatality rate hovering around 35% [4].

Fast forward to September 2022; the World Health Organization (WHO) has chronicled 2605 confirmed global instances of the MERS affliction, accompanied by 937 deaths. A significant fraction of these cases, 2196 to be precise, with 856 subsequent deaths, has been concentrated in Saudi Arabia. The year 2023 has already been witness to three verified MERS cases on an international scale, all pinpointed to Saudi Arabia, with two culminating in demise [5].

A closer scrutiny into the virulence mechanisms of MERS-CoV unveils the significance of certain viral proteins [6]. Prominently, the main protease, denoted as Mpro and synonymously referred to as 3C-like protease (3CLpro), has a cardinal function in the cleavage of viral polyproteins, pivotal for the virus’s replication and transcription endeavors [7]. GC376 and GC813 [8] are the known active inhibitors of MERS-CoV protease. These inhibitors target the enzyme named 3C-like protease, which is required for viral replication. These inhibitors are currently undergoing preclinical development. Acknowledging the centrality of Mpro in the virus’s operational machinery, it has been earmarked as a crucial molecular fulcrum for therapeutic interventions against MERS-CoV. Strategically thwarting Mpro’s activity offers a potential avenue to stifle the virus’s propagation trajectory, thereby attenuating the disease’s severity [9].

In the contemporary landscape of drug discovery, computational methodologies have come to the fore. Utilizing algorithms tailored for molecular interactions, such as molecular docking and dynamics simulations, researchers can now sift through vast molecular libraries [10]. The objective is to spotlight compounds that might form inhibitory liaisons with Mpro, thwarting its function. Notably, this digital paradigm trims both the temporal and fiscal investments associated with conventional drug discovery approaches [11]. For instance, Alamari and team discovered small covalent inhibitors from a database containing 28,790 compounds using pharmacophore modeling, covalent docking, and molecular dynamic simulation [12]. Similarly, Gyebi et al. used molecular docking techniques to identify alkaloids and terpenoids that find their binding affinity with MERS Mpro protein during their research on the identification of potential inhibitors of Mrpo or 3CL^pro^ [13]. Another piece of research conducted by Rahman and the team also identified three compounds from the ZINC database using a structure-based and ligand-based virtual screening process. Computational strategies have actively sought MERS-CoV Mpro antagonists, revealing potential therapeutic candidates. Despite progress, the ever-changing viral genomic landscape emphasizes the constant need for ongoing scientific exploration due to mutations and the emergence of drug-resistant strains [14].

Exploring aquatic environments, brown and green algae stand out as promising sources of bioactive compounds. With diverse chemical structures and functions among their numerous secondary metabolites, these algae are considered strong candidates for drug discovery. Over time, compounds derived from these algae have showcased antiviral capabilities against various pathogens [15,16]. Bioactive compounds derived from microalgae show promise for antiviral therapeutic development. Researchers have methodically assessed multiple microalgae strains, emphasizing the identification, extraction, isolation, and purification of novel bioactive molecules with essential therapeutic characteristics to combat viral infections [17].

Studies have proved that algae and cyanobacteria stand out as exceptionally rich sources of bioactive compounds endowed with potent antiviral properties and significant pharmacological activity. These microorganisms have garnered attention for their ability to produce a wide array of compounds that exhibit substantial potential in combating viral infections such as SARS-CoV-2 [18]. Phycocyanobilins (PCBs), allophycocyanin, lectins, and sulfated polysaccharides are among the key bioactive compounds sourced from various algal species. These compounds have demonstrated noteworthy therapeutic potential in addressing viral infections, including SARS-CoV-2, HIV, HSV, and MERS [19]. In this manuscript, we will delineate our computational exploration aimed at unearthing potential inhibitors of MERS-CoV Mpro sourced from marine brown algae. Our endeavors have been channeled towards assessing the binding propensities and interaction behavior of compounds from brown algae with Mpro. Through this research, we aim to contribute to the world’s efforts against MERS-CoV by leveraging the untapped molecular wealth nestled within marine algae.

## 2. Results

### 2.1. Virtual Screening, and Re-Docking

To find out the promising inhibitors against the MERS main protease by exploring the wide range of chemicals found in brown algae, a 3D configuration of MERS-CoV Mpro of PDB ID 8HUT was sourced from the Protein Data Bank (https://www.rcsb.org/, accessed on 10 May 2023) [20] and was prepared on the Chimera [21]. This computational journey was initiated by screening a vast library encompassing 1212 brown-algae-derived molecules which were downloaded from the Comprehensive Marine Natural Products Database (CMNPD) (https://www.cmnpd.org/, accessed on 10 May 2023) [22].

**Figure 1:**
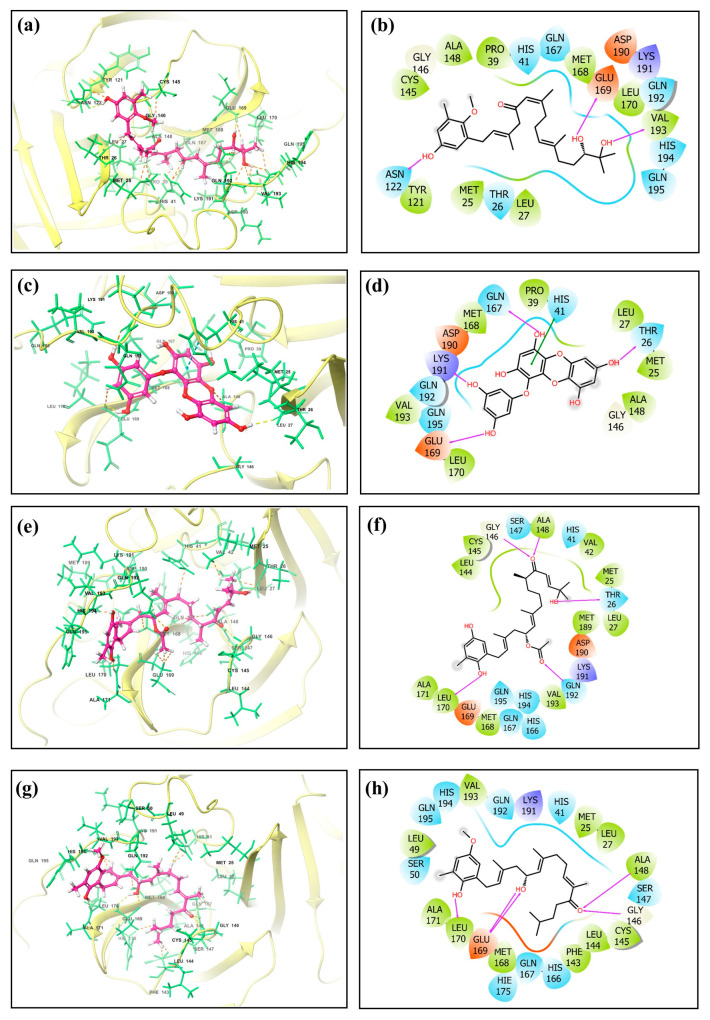
Three-dimensional and two-dimensional molecular interaction representation of ligands (**a**,**b**) CMNPD27819, (**c**,**d**) CMNPD1843, (**e**,**f**) CMNPD4184, and (**g**,**h**) CMNPD3156.

The screening results revealed noteworthy variations in the docking scores, which ranged from −0.11 to −8.6 kcal/mol. A list of the top 50 molecules, along with their docking scores, is given in Table S1. Four standout compounds, namely, CMNPD27819 (cystone C), CMNPD1843 (eckol), CMNPD4184, and CMNPD3156, were chosen based on their superior docking scores. This selection, however, was the tip of the computational iceberg. The free maestro visualization software [23,24] was used for generating the 3D and 2D images of the docked complexes (Figure 1 and Supplementary Figure S1).

The subsequent phase, re-docking using the Mti-Autodock tool, sought to augment the fidelity of our primary findings. 7YY, an experimentally validated inhibitor of the target protein, was taken for comparative study with hit molecules. CMNPD27819, CMNPD1843, CMNPD4184, and CMNPD3156 (−8.5 kcal/mol, −8.36 kcal/mol, −8.28 kcal/mol, and −8.25 kcal/mol) showed similar binding strengths and orientations to the reference molecule 7YY (−8.3 kcal/mol) during re-docking analysis. This supports their potential as strong inhibitors for MERS main protease. When we looked deeper into the molecular interactions, we could understand the specific details of these interactions (Table 1, Figure 1, and Supplementary Figure S1).

CMNPD27819 established pronounced specific interactions, e.g., hydrogen bonding with Glu^169^, Val^193^, and Asn^122^ and hydrophobic contact with Cys^145^, Gly^146,^ Ala^148^, Pro^39^, Met^168^, Leu^170^, Val^193^, Leu^27^, Met^25^, and Tyr^121^ residues, while CMNPD1843, despite its similar docking affinity, exhibited a distinct interaction pattern, predominantly hydrogen bonding with Thr^26^, Gln^167^, Lys^191^, and Glu^169^, hydrophobic contacts with Leu^170^, Val^193^, Met^168^, Pro^39^, Leu^27^, Met^25^, and Ala^148^, and one π–π stacking with His^41^. CMNPD4184 established hydrogen bonding with Gly^146^, Ala^148^, Thr^26^, Gln^192^, and Leu^170^ and hydrophobic contact with Leu^144^, Cys^145^, Ala^148^, Val^42^, Met^25^, Leu^27^, Met^189^, Val^193^, Met^168^, Leu^170^, and Ala^171^. CMNPD3156 showed hydrogen bonding with Ala^148^, Gly^146^, Glu^169^, and Leu^170^ and hydrophobic contacts with Leu^49^, Val^193^, Met^25^, Leu^27^, Ala^148^, Cys^145^, Leu^144^, Phe^143^, Met^168^, Leu^170^, and Ala^171^. The reference molecule showed hydrogen bonding with Thr^26^, Ala^148^, Ser^147^, Gly^146^, and Glu^169^ and hydrophobic contacts with Phe^143^, Leu^144^, Cys^145^, Ala^148^, Leu^27^, Met^25^, Tyr^54^, Val^42^, and Met^168^. One π–π stacking was also observed for the reference molecule with His^41^ residue (Table 1, Figure 1, and Supplementary Figure S1).

This interaction behavior underscores the intricate nature of enzyme–ligand interplay and the need for a comprehensive understanding of such interactions for drug design. Entirely, this exhaustive in silico voyage accentuates brown algae’s untapped potential in the realm of antiviral therapeutics. The convergence of virtual screening, re-docking, and detailed interaction analytics has bequeathed a promising set of candidates poised for subsequent experimental validations. However, the odyssey from digital insights to tangible therapeutic agents is extensive and demands rigorous empirical studies. While CMNPD27819, CMNPD1843, CMNPD4184, and CMNPD3156 have emerged as frontrunners in this digital milieu, their real-world antiviral efficacy remains to be ascertained. The initial successes documented herein are promising, but they also underscore the vast, multifaceted journey ahead in the quest for efficacious MERS therapeutics.

### 2.2. Molecular Dynamics Simulations

**Figure 2:**
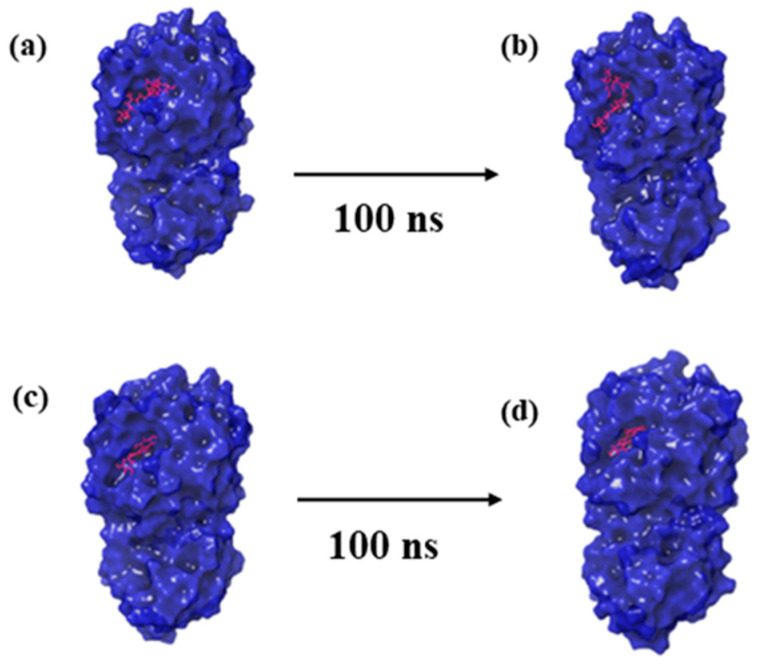

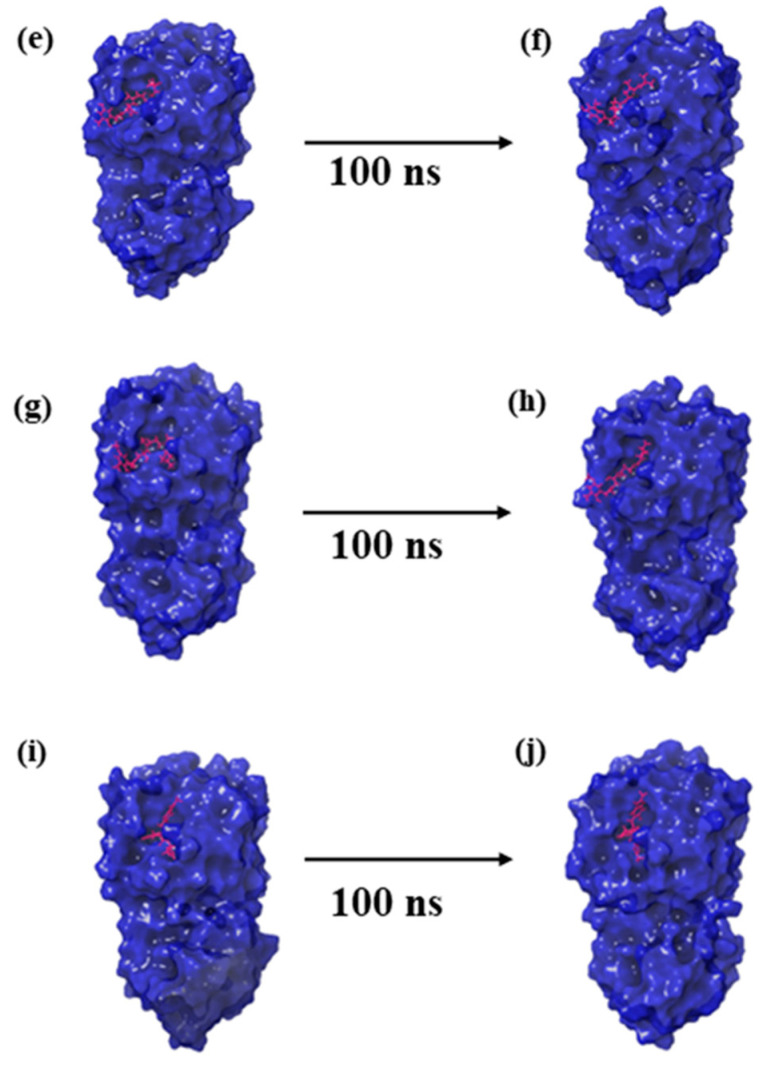
Three-dimensional structural representation of first and last poses of MERS protease docked complexes, i.e., (**a**,**b**) protease–CMNPD27819 complex, (**c**,**d**) protease–CMNPD1843 complex, (**e**,**f**) protease–CMNPD4184 complex, (**g**,**h**) protease–CMNPD3156 complex, and (**i**,**j**) protease–reference complex obtained from simulation trajectory.

Molecular dynamics (MD) simulations offer a temporal lens to apprehend the stability and dynamics of protein–ligand complexes [23]. To understand how CMNPD27819, CMNPD1843, CMNPD4184, and CMNPD3156 bind to the MERS main protease, we conducted a detailed MD simulation study on the results with the control molecule, 7YY. The first and last pose of each protein–ligand complex obtained from the MD simulation trajectory are represented in Figure 2.

#### 2.2.1. Root-Mean-Square Deviation (RMSD)

**Figure 3:**
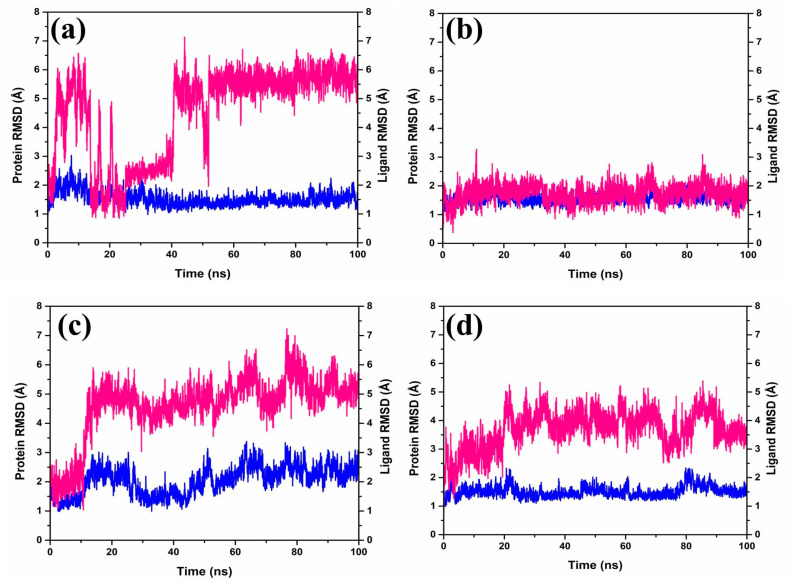
RMSD plots of the MERS protease docked complexes, i.e., (**a**) protease–CMNPD27819 complex, (**b**) protease–CMNPD1843 complex, (**c**) protease–CMNPD4184 complex, and (**d**) protease–CMNPD3156 complex generated from 100 ns simulation trajectories. Here, the protein and ligand are represented by the blue and red lines, respectively.

The Root-Mean-Square Deviation (RMSD) (Figure 3) plays a role in quantifying the magnitude and characteristics of the spatial alterations observed within protein–ligand pairs following their combination into complexes. Upon diving into the RMSD data (Figure 3) generated by the software Desmond-maestro [24,25], an interesting trajectory was observed for the protease–CMNPD27819 complex. This trajectory initially was unstable at up to 40 ns (Figure 3a), then exhibited fluctuations reaching 6–7 Å, and finally stabilized around an equilibrium of approximately 6.5 Å during the later stages of the simulation (60–100 ns). Remarkably, the protein remained anchored in its binding niche. For the protein–ligand complex, there was a noticeable RMSD shift of about 3 Å early on, yet it retained its position in the protein’s active binding site, with distances to the interacting ligands consistently below 6.5 Å for the duration of the study.

Shifting our focus to the protease–CMNPD1843 combination, it displayed admirable consistency, with RMSD values hovering between 2 and 3 Å over the entire 100 ns simulation span (Figure 3b). In comparison, the protease–CMNPD4184 complex demonstrated noticeable RMSD shifts within 2–4 Å for the initial 20 ns (Figure 3c). Post this phase, ligand RMSD values settled between 3 and 5 Å up to the completion of the 100 ns simulation, while the protein’s RMSD displayed variations within the 2–3 Å range. Likewise, the protease–CMNPD3156 complex showcased distinct RMSD patterns, with the ligand starting with a deviation around 2 Å, persisting up to 15 ns (Figure 3d), followed by prominent fluctuations ranging between 5 and 8 Å. Simultaneously, protein-centric RMSD readings highlighted variations consistent with ligand adjustments, ranging from 1–3 Å.

However, the protein in the protease–7YY reference complex exhibited stable RMSD between 2 and 3 Å. Correspondingly, the ligand displayed RMSD shifts of up to 2–3 Å, hinting at a dynamic relationship with the receptor protein (Supplementary Figure S2).

Based on our thorough assessments, a key finding stands out; the CMNPD1843 compound stands out for its exceptional stability compared to other compounds derived from the brown marine algae. This highlights the potential effectiveness of compounds from marine sources for therapeutic purposes. Essentially, these simulations unveil the intricate yet strong interplay between these molecules, providing insights into the complex interactions between ligands and proteins.

#### 2.2.2. Root-Mean-Square Fluctuation (RMSF)

Root-Mean-Square Fluctuation (RMSF) provides a vivid depiction of atomic flexibility within protein structures during molecular dynamics (MD) simulations, serving as a proxy for identifying regions of protein variability upon ligand binding. When delving deeply into the RMSF of our carefully selected natural compound structures, a consistent theme of moderate variability became evident throughout the duration of the simulation. Interestingly, a recurring pattern was discernible in the C-terminal residues, specifically within the 0–150 range, across the vast majority of these assemblies. A notable exception to this was the CMNPD1843 complex, where two significant spikes surpassed 3 Å. In alignment with this observation, our benchmark protein mirrored similar variabilities.

Looking at the RMSF (Root-Mean-Square Fluctuation) of all the paired ligands, including our reference 7YY, we noticed a good level of stability. The fluctuations were quite small, staying below 4 Å throughout the entire MD simulation (Supplementary Figure S3). When we checked the ligand RMSF in these pairs, it confirmed that the ligands stayed well placed in the active pocket with only minor changes, around 1–1.5 Å in the atomic structures of the ligands, compared to the protease protein. This pattern is similar to what we saw with our reference compound. Importantly, these observed measurements match the RMSF trends shown by our reference compound (Supplementary Figure S4). While CMNPD1843 closely mirrored the control, offering promise, the diverse RMSF landscapes painted by others serve as a testament to the intricate dance of protein–ligand dynamics and the imperative nature of its comprehensive understanding in drug discovery.

#### 2.2.3. Protein–Ligand Contact Analysis

**Figure 4:**
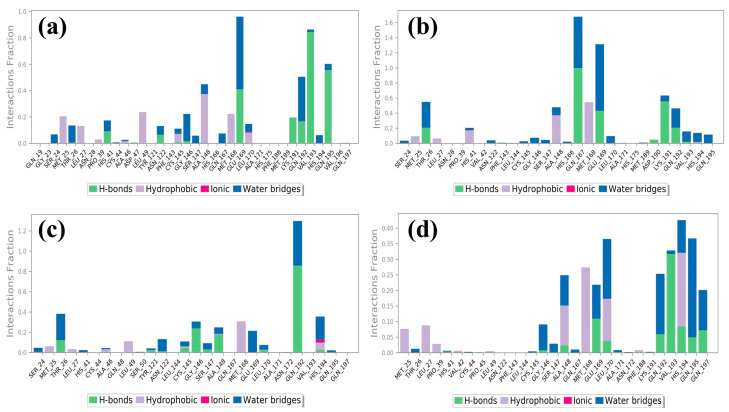
Protein–ligand contacts of MERS protease docked complexes, i.e., (**a**) protease–CMNPD27819 complex, (**b**) protease–CMNPD1843 complex, (**c**) protease–CMNPD4184 complex, and (**d**) protease–CMNPD3156 complex formed during the 100 ns simulation.

Molecular dynamics simulations help us see how ligands interact with proteins, focusing on things like hydrogen bonding and hydrophobic interactions over time [26]. In the case of the protease–CMNPD27819 assembly (Figure 4a), before the simulation started, we identified residues like Asn122, Glu169, and Val193 as forming three separate hydrogen bonds with their neighboring parts. To understand how the ligand binds in the protein, we looked at both hydrogen bonding and hydrophobic interactions throughout the simulation. During the simulation, the Asn122 residue changed its interaction, while both Glu169 and Val193 continued to form hydrogen bonds. They interacted with the protein for about 36% and 72% of the entire simulation, respectively. At the same time, new hydrogen bonds appeared, especially with Gln195, making up 52% of the interactions. This shift in residues and the emergence of new bonds show how ligand–protein interactions can adapt and change during engagement. Notably, the Ala148 residue showed hydrophobic bonding interactions. In the initial docking interactions, we saw residues Thr26, Gln167, and Glu169 forming hydrogen bonds with the ligand. But, in later molecular dynamics simulations, there was a switch from Thr26 to Lys191, and His41 showed a pi–pi cation interaction. For the docked protease–CMNPD1843 (Figure 4b), hydrogen bonds were present 99%, 49%, and 36% of the time with residues Gln167, Lys191, and Glu169, respectively. This indicates the strong stability of these complexes.

**Figure 5:**
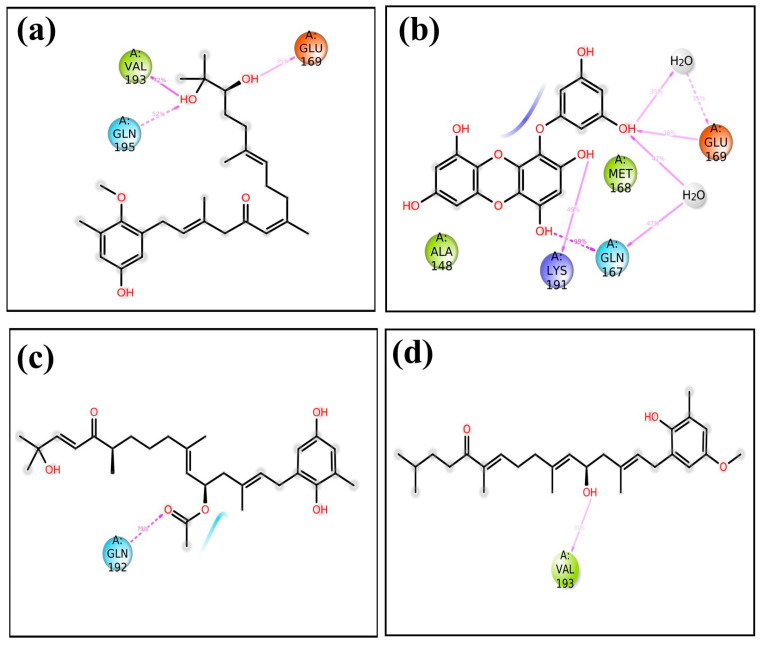
Schematic representation of protein residues’ contacts with ligand for more than 30% of simulation time of MERS protease docked complexes, i.e., (**a**) protease–CMNPD27819 complex, (**b**) protease–CMNPD1843 complex, (**c**) protease–CMNPD4184 complex, (**d**) protease–CMNPD3156 complex.

Shifting our focus to the protease–CMNPD4184 complex (Figure 4c), initial docking interactions showcased residues like Thr26, Gly146, Ala148, Leu170, and Gln192 forming hydrogen bonds. Notably, the Gln192 residue consistently maintained hydrogen bond formation during simulation, accounting for over 79% of the bonding interactions. The His194 residue also demonstrated a noteworthy pi–pi interaction over the simulation period. For the protease–CMNPD3156 complex (Figure 4d), initial docking pinpointed residues Gly146, Ala148, Glu169, and Leu170 as forming hydrogen bonds. However, as the simulation progressed, some of these bonds dissipated, with Val193 surfacing and accounting for 31% of the interactions across the 100 ns simulation window (Table S2 and Figure 1, Figure 4 and Figure 5).

Further, when observing the interactions between the MERS protease and reference molecule 7YY, hydrogen bond formation was detected with residues Thr26, Gly146, Ser147, Ala148, and Glu169 (Supplementary Figures S5 and S6 and Table S2). Concurrently, His41 was observed forming a pi–cation bond. These interactions remained consistent during the simulation. The interaction of the reference compound 7YY with MERS protease yielded significant hydrogen bond interactions, with residues Thr26, Gly146, Ser147, Ala148, Glu169, and His175 displaying occupancies of 99%, 82%, 44%, 67%, 84%, and 74%, respectively, throughout the 100 ns simulation. The His41 residue was observed engaging in hydrophobic bonding interactions. These interaction percentages, along with additional observations, highlight the inherent stability of the complexes and offer comparable insights with respect to the studied compounds (Figure 1, Figure 4, and Figure 5, Table 1, and Supplementary Table S2).

### 2.3. Principal Components Analysis (PCA)

Principal Component Analysis (PCA) is a technique widely utilized to simplify the multitudinous dimensions seen in molecular dynamics (MD) simulation trajectories. Through this method, a collective observation of atomic shifts across all coordinates can be achieved while simultaneously deriving vital statistical data. A closer look at the initial three principal components serves to unpack and provide clarity on the intricate nuances of atomic displacements.

**Figure 6:**
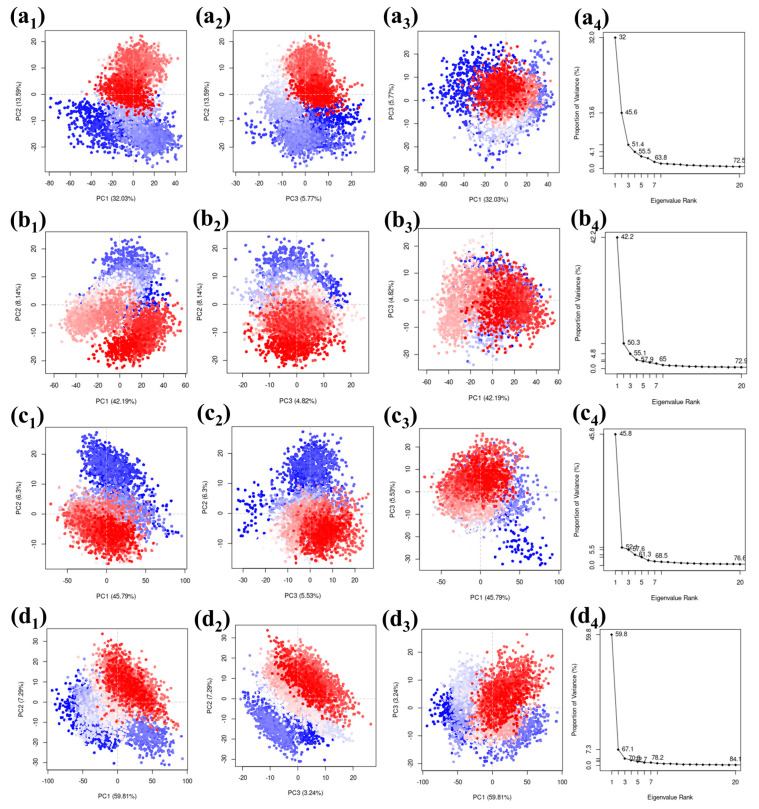
Scatter plots and scree plots obtained from the PCA analysis of the MERS protease docked complexes, i.e., (**a1**–**a4**) protease–CMNPD27819 complex, (**b1**–**b4**) protease–CMNPD1843 complex, (**c1**–**c4**) protease–CMNPD4184 complex, and (**d1**–**d4**) protease–CMNPD3156 complex.

We used a PCA (Principal Component Analysis) method on a carefully selected group of complexes. The results were visually presented, focusing on the top three principal components represented as PC1, PC2, and PC3. The eigenvalues associated with these components represented how much of the variability in the simulation of these compounds can be attributed to each component. When looking at the protease–CMNPD27819 combination, we found that the initial three principal components explained a substantial 51.39% of the overall variance. This percentage gives us insights into how the compound’s structure behaves. Similarly, for the protease–CMNPD1843 combination, these components accounted for 55.15% of the variance, emphasizing their importance in understanding structural changes. The PCA results for protease–CMNPD4184 showed 57.62% of the overall variance. Remarkably, protease–CMNPD3156 exhibited a total variance of 70.34% in its three main components, indicating significant structural changes during the simulation in the docked molecular structure. The visuals show the docked entities alongside their eigenvalues, revealing the percentages of variance linked to the main three components (Figure 6). The reference molecule 7YY was responsible for covering 46.01% of the data representation (Figure S7).

Divergent clusters in the scattered plots—differentiated through shades of white, blue, and red—denote configurational shifts in the docked ensemble. It demonstrates considerable congruency between the PC1 and PC2, PC2 and PC3, and PC1 and PC3 plots across the quadruple coordinates, which is indicative of the molecule’s structural fluctuations in the particular dimensions. The overlapping of the three colors is seen in the scattered plots of all the complexes, including the reference complex. This observation states that a similar configuration of the complex was observed for the maximum number of times during the simulation. This also confirms that no confirmational changes were observed in the protein or ligand. The complex was stable during the simulation phase. The scree plots obtained based on the comparative scatterplot show a steep slope for all the complexes. This further confirms that no confirmation changes were observed in the complex during the simulation. These findings may suggest that the selected ligand may pose the inhibitory function against the target protein due the stable binding state and absence of confirmational changes (Figure 6).

## 3. Discussion

The voyage into the diverse chemical landscape of brown-algae-derived molecules offers a promising avenue in the quest for potential MERS main protease inhibitors. Brown algae’s longstanding reputation for housing compounds with multifaceted therapeutic traits has been well documented [27]. By exploring this untapped reservoir of bioactive molecules, our computational exploration unearthed four potential inhibitors that exhibited remarkable binding affinities, akin to a native inhibitor [28].

Re-docking studies, although often underestimated, play a pivotal role in confirming the reliability of the top contenders. In this context, the almost synonymous binding affinities of the identified compounds and the control molecule accentuate their potential as MERS inhibitors. Such comparative analyses are vital for drug design, as it stresses the significance of not just binding affinities, but also the specificity of molecular interactions that underlie these affinities [29]. As observed, even though these compounds, CMNPD27819, CMNPD1843, CMNPD4184, and CMNPD3156, exhibit similar docking affinities, their interaction patterns with the protease reveal stark differences, underscoring the intricate nature of enzyme–ligand interplay.

Molecular dynamics simulations, a cornerstone for assessing the stability and dynamism of protein–ligand complexes, painted a vivid picture of our candidate compounds [30]. The RMSD trajectory, an instrumental parameter for elucidating the spatial adjustments of complexes, demonstrated that our algae-derived compounds, especially CMNPD1843, displayed pronounced stability during the simulations compared to the reference molecule, 7YY. Such stability is indicative of a potential therapeutic application, especially when considering the dynamic environment of cellular matrices where these compounds will eventually function [31].

Further, the RMSF value, a proxy for atomic flexibility, remained consistent for our compounds, reinforcing their potential stability in biological systems [32]. Interestingly, CMNPD1843′s behavior closely emulated the reference molecule, underscoring its potential as a frontrunner in this explorative race.

Protein–ligand contact analysis delved deep into the nuances of molecular engagement, especially hydrogen bonding and hydrophobic interactions. The evolving nature of ligand responsiveness to the protein milieu during engagement was particularly evident in compounds such as CMNPD27819 and CMNPD1843, which displayed dynamic shifts in hydrogen bond formations over the simulation period. This adaptive nature is instrumental for therapeutic agents as it ensures their efficacy in dynamic physiological environments [33,34].

Principal Components Analysis (PCA), a cornerstone for simplifying MD trajectories, was invaluable in deciphering the complex dance of atomic displacements in our compounds [35]. The significant variance captured by the principal components, especially for the protease–CMNPD27819 ensemble, accentuates the dynamic conformational changes these compounds undergo, which are paramount for their biological functionality.

This exhaustive computational journey underscores the immense potential brown algae compounds hold in the realm of antiviral therapeutics. While the in silico insights are promising, the journey from the digital realm to tangible therapeutic agents is vast and warrants rigorous empirical validation. The compounds CMNPD27819, CMNPD1843, CMNPD4184, and CMNPD3156 stand out as promising candidates. However, their real-world antiviral efficacy remains an open question.

## 4. Materials and Methods

### 4.1. Data Collection, Virtual Screening, and Re-Docking

The structure of the MERS main protease, identified with the PDB ID 8HUT (https://www.rcsb.org/versions/8HUT, accessed on 10 May 2023), was sourced from the Protein Data Bank [20]. Prior to virtual screening, any co-crystallized ligands, water molecules, or other heteroatoms were removed, and the protein structure was subjected to energy minimization. The crystallized ligand 7YY (PubChem CID: 162533924) in the binding pocket of the 8HUT was taken as the reference molecule for the comparative study with hit compounds. A sum of 1212 bioactive compounds sourced from phaeophyceae, especially brown algae (organism ID—CMNPD330064) (https://www.cmnpd.org/organism-report-card/CMNPD330064, accessed on 10 May 2023), were retrieved from the compound summaries accessible in the Comprehensive Marine Natural Products Database (CMNPD) (https://www.cmnpd.org/, accessed on 10 May 2023) [22].

The compounds were screened against the optimized MERS main protease structure using the MTiOpenScreen platform. The platform provides an interactive and streamlined virtual screening approach, allowing for a robust identification of potential ligand–protein interactions. Default parameters provided by the MTiOpenScreen platform were employed [36]. The compounds were automatically pre-processed, which included the calculation of charge states and the optimization of their 3D conformations. The center coordinates and grid box size were provided for screening of molecules within the active site residue region. To further verify the results obtained from the initial virtual screening, the top-ranking compounds, based on binding scores and predicted interactions, were chosen for re-docking. These selected ligands underwent an additional pre-processing phase to ensure their readiness for the re-docking procedure. Using MTi-Autodock, the selected compounds were re-docked into the active site of the MERS main protease. MTi-Autodock offers a refined and detailed analysis of ligand–protein interactions, providing insights into binding orientations and potential interaction hotspots. The software’s flexible receptor docking approach was utilized to account for the flexibility of the protein and ligand during the binding event [37].

### 4.2. Molecular Dynamics Simulation

To delve deeper into the intricate molecular behavior of the MERS main protease in interaction with potential inhibitors from brown algae, we executed comprehensive molecular dynamics (MD) simulations.

#### 4.2.1. System Building and Minimization

Our study incorporated a set of five complexes, which encompassed four unique protein–ligand structures and one reference complex, each poised for MD simulation via Desmond [24,25]. Initially, every complex was immersed in an orthorhombic simulation box and populated with the TIP4P water model, ensuring a minimum buffer of 10 Å from the protein to the box boundaries [38,39]. This setup promotes spontaneous molecular movement within a hydrated environment that approximates physiological conditions. The OPLS-2005 force field was chosen for its proven reliability in capturing the nuances of protein–ligand interactions [40,41]. OPLS-2005 has less accuracy with the broad data. OPLS-2005 is frequently used in molecular mechanics simulations to model organic molecules, proteins, and nucleic acids and behavioral study due its improved accuracy compared to the previous version [42].

#### 4.2.2. MD Simulation Execution

After the initial setup, the systems started a lengthy 100 ns MD simulation run. The simulations were operated under canonical ensemble (NPT) conditions, with temperature and pressure being meticulously maintained at 300 K and 1 atm, respectively, using the Nosé–Hoover thermostat and the Martyna–Tobias–Klein barostat [43]. Periodic boundary conditions were applied in all three dimensions to simulate an infinite system. The simulations leveraged a time step of 2.0 fs with particle mesh Ewald (PME) for long-range electrostatics [44].

### 4.3. Principal Component Analysis

Utilizing the bio3d package within R, we conducted Principal Component Analysis (PCA) on the individual complexes sourced from our MD simulation trajectories [45]. PCA serves as a potent statistical tool to distill and highlight the pronounced residue fluctuations within the protein. By focusing on the alpha-carbon (Cα) atoms within the protein, we developed a covariance matrix which allowed us to grasp the primary motions. The eigenvectors, when ranked by their corresponding eigenvalues, provide us with what we term ‘principal components’. For our analysis, we emphasized the top three principal components, which typically capture the most significant dynamic behaviors. This analytical approach empowered us to deeply understand the protein’s motion dynamics through the computed principal components.

## 5. Conclusions

The current study hinged on the computational exploration of brown algae compounds to discern potential inhibitors against the MERS main protease. Our meticulous virtual screening and re-docking endeavors resulted in four standout candidates: CMNPD27819, CMNPD1843, CMNPD4184, and CMNPD3156. These compounds, bolstered by their affinities mirroring a native inhibitor, substantiated their potential as formidable MERS inhibitors. Molecular dynamics simulations further provided an enlightening perspective on the stability and intricate dynamics of these protein–ligand interactions. Notably, CMNPD1843 exhibited unparalleled stability during MD simulation sessions, distinguishing it from its marine-derived counterparts.

RMSD and RMSF analyses, coupled with a detailed protein–ligand contact study, reinforced the stability and dynamic interplay of these molecular entities. Furthermore, Principal Component Analysis (PCA) facilitated the discernment of atomic displacements and emphasized the compounds’ structural behaviors relative to the native inhibitor.

In essence, our in silico investigations have underlined the unexplored antiviral potential of brown-algae-derived compounds. In this study, we wish to underscore the novelty of our research by explicitly stating that the four compounds, CMNPD27819 (cystone C), CMNPD1843 (eckol), CMNPD4184, and CMNPD3156, have not been previously examined, whether through laboratory experiments (in vitro/in vivo) or computational modeling (in silico), for their impact on MERS-CoV protease or any other coronaviruses. This research represents the first investigation into the potential effects of these compounds on these viral targets, contributing to our understanding of their antiviral properties.

## Figures and Tables

**Table 1:** List of interactions with their respective interacting residues.

S. No.	Complex	H Bond	Hydrophobic	π–π Stacking/ π–π Cation *
**1**	MERS protease–CMNPD27819	Glu^169^, Val^193^, Asn^122^	Cys^145^, Gly^146^, Ala^148^, Pro^39^, Met^168^, Leu^170^, Val^193^, Leu^27^, Met^25^, Tyr^121^	--
**2**	MERS protease–CMNPD1843	Thr^26^, Gln^167^, Lys^191^, Glu^169^	Leu^170^, Val^193^, Met^168^, Pro^39^, Leu^27^, Met^25^, Ala^148^	His^41^
**3**	MERS protease–CMNPD4184	Gly^146^, Ala^148^, Thr^26^, Gln^192^, Leu^170^	Leu^144^, Cys^145^, Ala^148^, Val^42^, Met^25^, Leu^27^, Met^189^, Val^193^, Met^168^, Leu^170^, Ala^171^	--
**4**	MERS protease–CMNPD3156	Ala^148^, Gly^146^, Glu^169^(2), Leu^170^	Leu^49^, Val^193^, Met^25^, Leu^27^, Ala^148^, Cys^145^, Leu^144^, Phe^143^, Met^168^, Leu^170^, Ala^171^	--
**5**	MERS protease–control (7YY)	Thr^26^, Ala^148^, Ser^147^, Gly^146^, Glu^169^	Phe^143^, Leu^144^, Cys^145^, Ala^148^, Leu^27^, Met^25^, Tyr^54^, Val^42^, Met^168^	His^41^

* mark indicates that the * π–cation is the interaction involved in the post-dynamic analysis reference complex.

## Data Availability

The datasets used and analyzed during the current study are available from the corresponding author at reasonable request.

## Supplementary Materials

The following are available online at https://www.mdpi.com/article/10.3390/md21120626/s1: Table S1: List of top 20 screened compounds and their docking energy; Table S2: Intermolecular interaction for the selected compound post dynamic analysis of MERS protease. * mark indicate the * π- cation is the interaction involved in the post dynamic analysis reference complex; Figure S1: 3D and 2D interaction diagram of protein ligand interaction of MERS protease with control molecule 7YY; Figure S2: RMSD value resulting from MERS protease with control molecule-7YY during molecular dynamics simulation over 100 ns; Figure S3: The protein root mean square fluctuation (P-RMSF) of docked protein-ligand complexes during 100 ns simulation: (a) Protease-CMNPD27819 complex, (b) Protease-CMNPD1843 complex (c) protease-CMNPD4184 complex, (d) protease-CMNPD3156 complex, and (e) protease-reference/control complex; Figure S4: The Ligand root mean square fluctuation (L-RMSF) of docked protein-ligand complexes during 100 ns simulation: (a) Protease-CMNPD27819 complex, (b) Protease-CMNPD1843 complex (c) protease-CMNPD4184 complex, (d) protease-CMNPD3156 complex, and (e) protease- reference/control complex; Figure S5: Protein-ligand interactions contact mapping of MERS protease with control molecule during 100 ns simulation; Figure S6: The ligand-protein contact of MERS protease with control molecule during 100 ns simulation; Figure S7: Principal component analysis for the generated for the reference molecule with docked MERS-protease protein target.
