# Supplementary material for: A Multifaceted Computational Approach to Understanding the MERS-CoV Main Protease and Brown Algae Compounds’ Interaction
Source: Mar Drugs. 2023 Nov 30;21(12):626. doi: 10.3390/md21120626 (PMC10744363; doi:10.3390/md21120626)
Supplement: Supplementary file 1 [file marinedrugs-21-00626-s001.zip › marinedrugs-2692037-supplementary.pdf]

S.N.	Title	Docking Energy
1.	CMNPD27819	-8.6
2.	CMNPD1843	-8.36
3.	CMNPD4184	-8.29
4.	CMNPD3156	-8.26
5.	CMNPD22358	-8.25
6.	CMNPD4742	-8.23
7.	CMNPD4739	-8.22
8.	CMNPD1845	-8.19
9.	CMNPD23661	-8.16
10.	CMNPD1826	-7.98
11.	CMNPD8828	-7.97
12.	CMNPD4740	-7.96
13.	CMNPD17787	-7.95
14.	CMNPD1842	-7.89
15.	CMNPD4735	-7.88
16.	CMNPD21103	-7.86
17.	CMNPD2218	-7.84
18.	CMNPD447	-7.81
19.	CMNPD9489	-7.81
20.	CMNPD17806	-7.77
21.	CMNPD21101	-7.74
22.	CMNPD17807	-7.74
23.	CMNPD427	-7.71
24.	CMNPD5924	-7.7
25.	CMNPD26471	-7.68
26.	CMNPD17788	-7.65
27.	CMNPD27816	-7.62
28.	CMNPD433	-7.56
29.	CMNPD4733	-7.56
30.	CMNPD3668	-7.55
31.	CMNPD2605	-7.55
32.	CMNPD23660	-7.54
33.	CMNPD5366	-7.51
34.	CMNPD5923	-7.49
35.	CMNPD19974	-7.49
36.	CMNPD3667	-7.46
37.	CMNPD8048	-7.45
38.	CMNPD4181	-7.44
39.	CMNPD3662	-7.42
40.	CMNPD5377	-7.4
41.	CMNPD26472	-7.35
42.	CMNPD5922	-7.35
43.	CMNPD4732	-7.27
44.	CMNPD23659	-7.26
45.	CMNPD1844	-7.25
46.	CMNPD14000	-7.22
47.	CMNPD4183	-7.21
48.	CMNPD4738	-7.18
49.	CMNPD4734	-7.17
50.	CMNPD4731	-7.14

Table S1- List of top 20 screened compounds and their docking energy

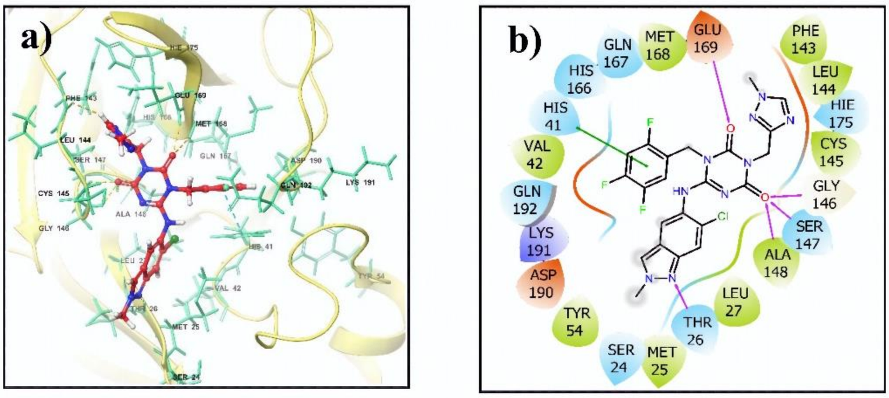

Figure S1- 3D and 2D interaction diagram of protein ligand interaction of MERS protease with control molecule 7YY.

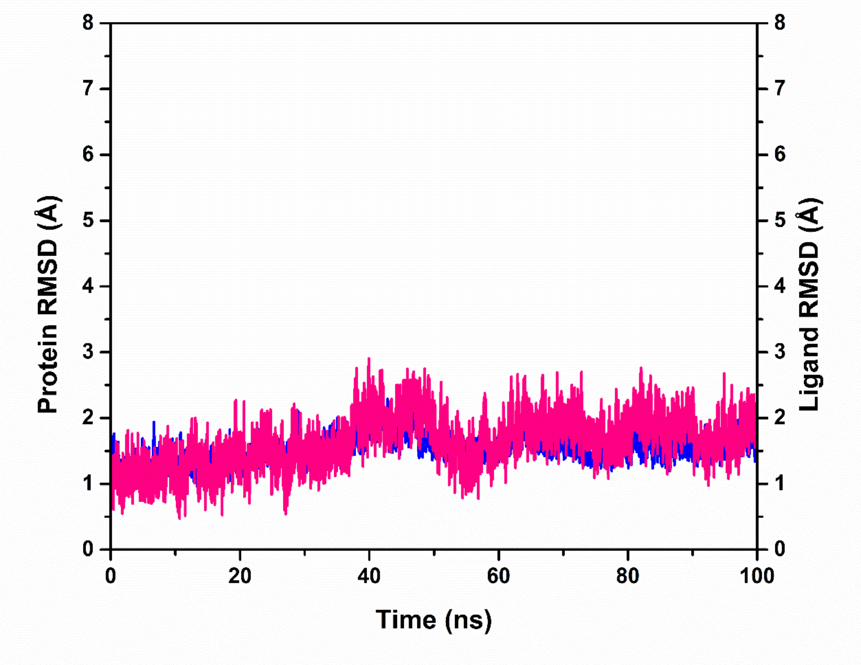

Figure S2- RMSD value resulting from MERS protease with control molecule-7YY during molecular dynamics simulation over 100 ns.

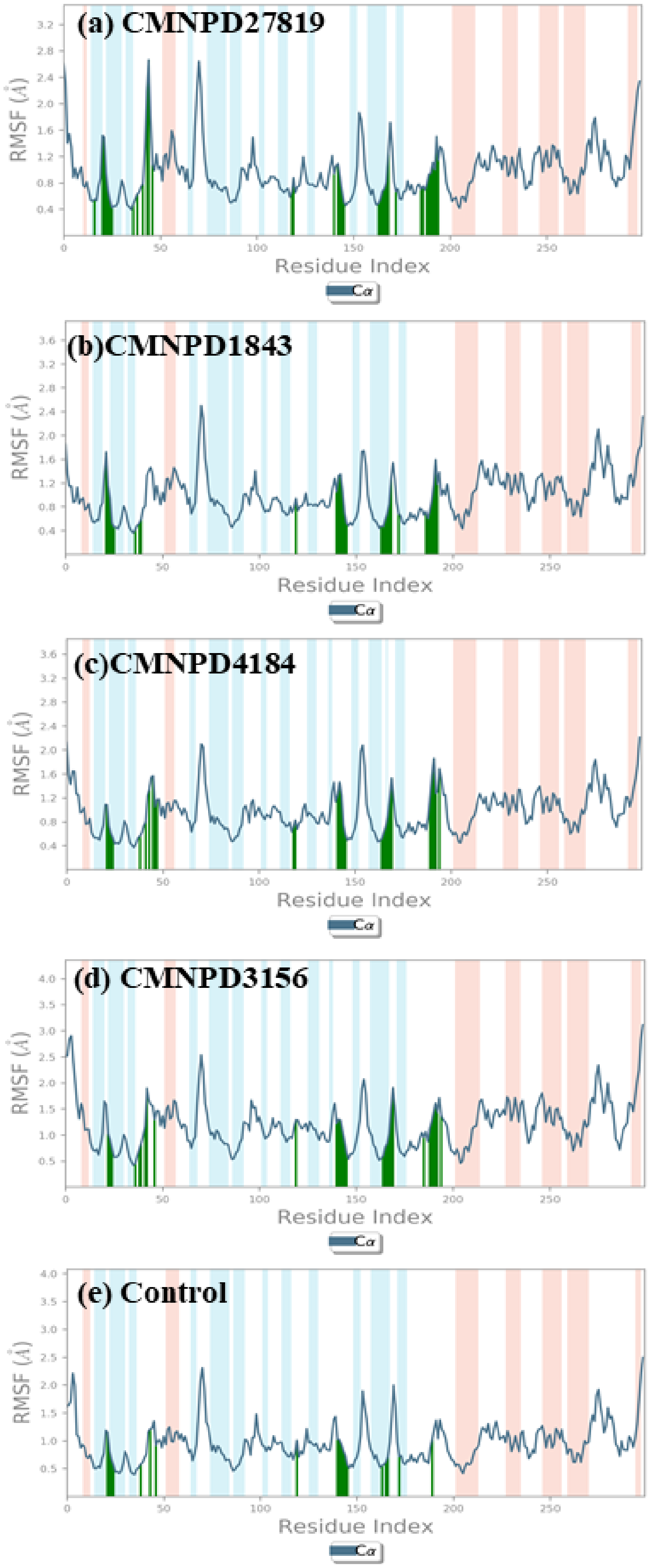

(a) CMNPD27819
RMSF (Å)
Residue Index
Cα
(b)CMNPD1843
RMSF (Å)
Residue Index
Cα
(c)CMNPD4184
RMSF (Å)
Residue Index
Cα
(d) CMNPD3156
RMSF (Å)
Residue Index
Cα
(e) Control
RMSF (Å)
Residue Index
Cα

Figure S3- The protein root mean square fluctuation (P-RMSF) of docked protein-ligand complexes during 100ns simulation: a) Protease-CMNPD27819 complex, b) Protease-CMNPD1843 complex c) protease-CMNPD4184 complex, d) protease-CMNPD3156 complex, and e) protease-reference/control complex.

(a) CMNPD27819

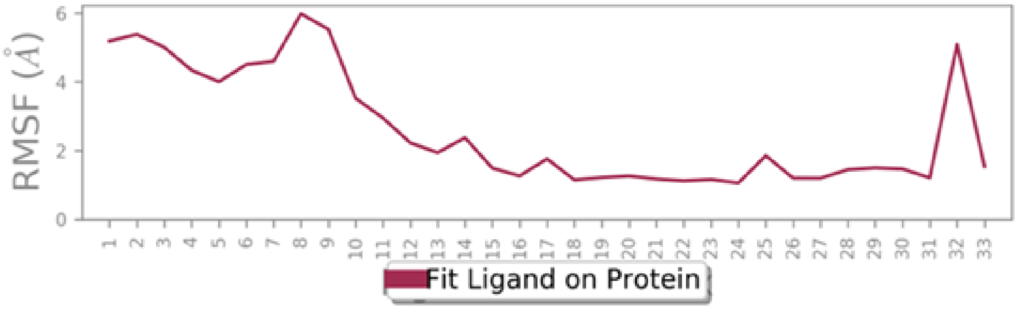

(b) CMNPD1843

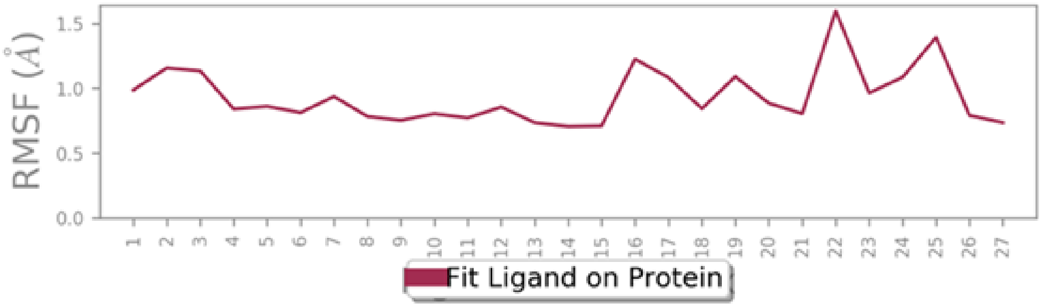

(c) CMNPD4184

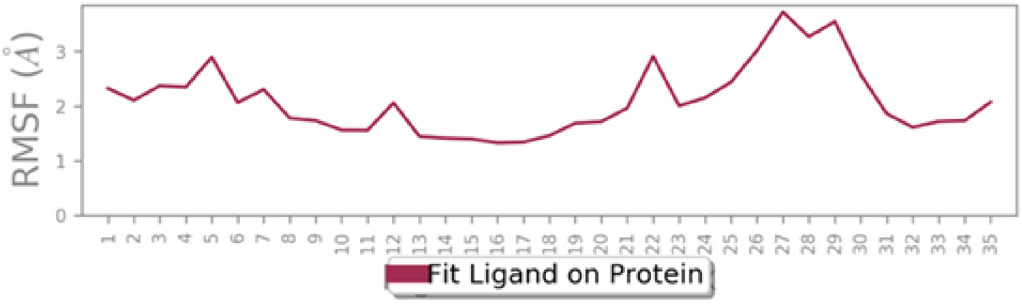

(d) CMNPD3156

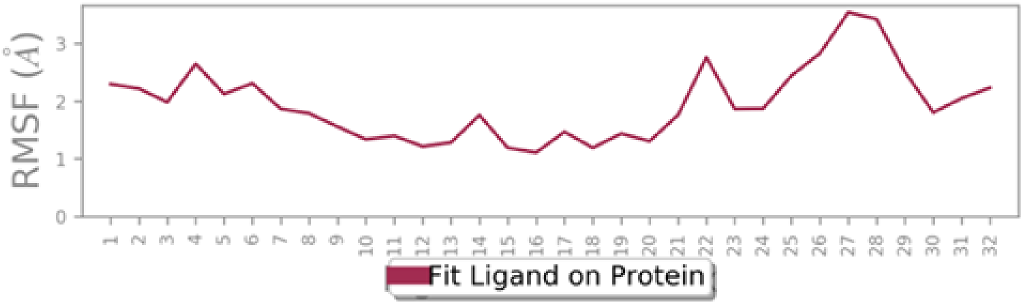

(e) Control

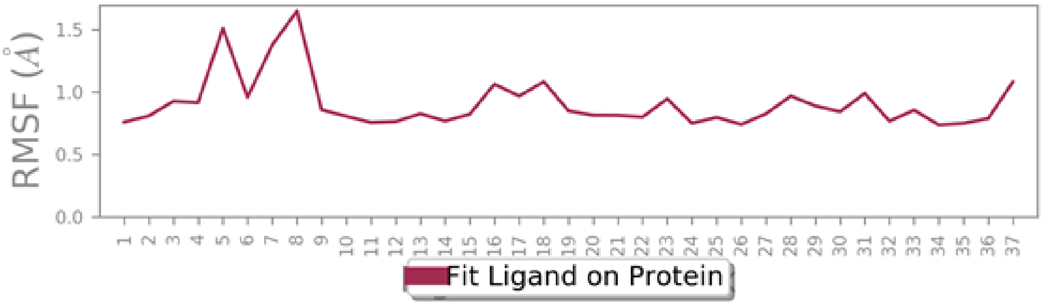

Figure S4- The Ligand root mean square fluctuation (L-RMSF) of docked protein-ligand complexes during 100ns simulation: a) Protease-CMNPD27819 complex, b) Protease-CMNPD1843 complex c) protease-CMNPD4184 complex, d) protease-CMNPD3156 complex, and e) protease- reference/control complex.

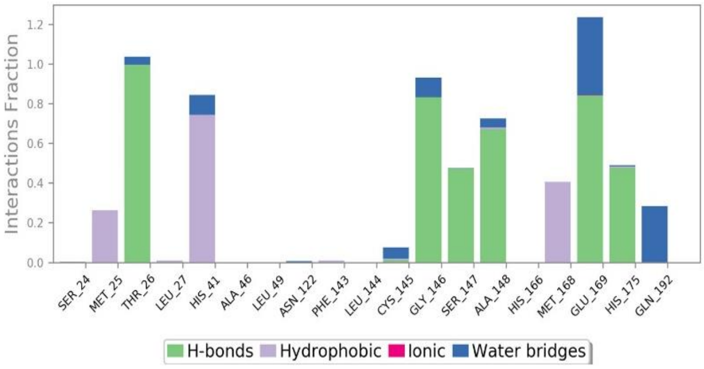

Figure S5- Protein-ligand interactions contact mapping of MERS protease with control molecule during 100ns simulation.

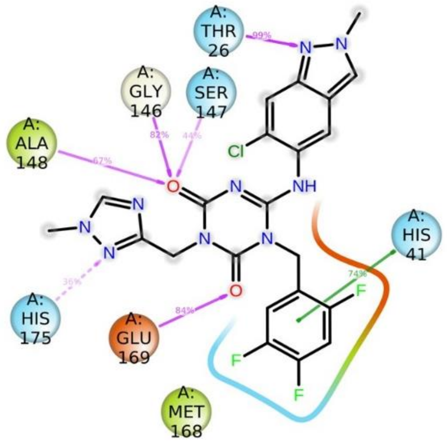

Figure S6- The ligand-protein contact of MERS protease with control molecule during 100ns simulation.

S. no.	Complex	H-Bond	Hydrophobic	**π-π stacking/ π-π cation***
1	MERS protease-CMNPD27819	Val^{193}, Gln^{195}, Glu^{169}	Val^{193}	--
2	MERS protease -CMNPD1843	Glu^{169}, Gln^{167}, Lys^{191}	Met^{169}, Ala^{148}	--
3	MERS protease-CMNPD4184	Gln^{192}	--	--
4	MERS protease-CMNPD3156	Val^{193}	Val^{193}	--
5	MERS protease-Control (7YY)	Glu^{169}, His^{175}, Ala^{148}, Gly^{146}, Ser^{147}, Thr^{26}	Ala^{148}, Met^{168}	His^{41}

Table S2 - Intermolecular interaction for the selected compound post dynamic analysis of MERS protease. * mark indicate the * π- cation is the interaction involved in the post dynamic analysis reference complex.

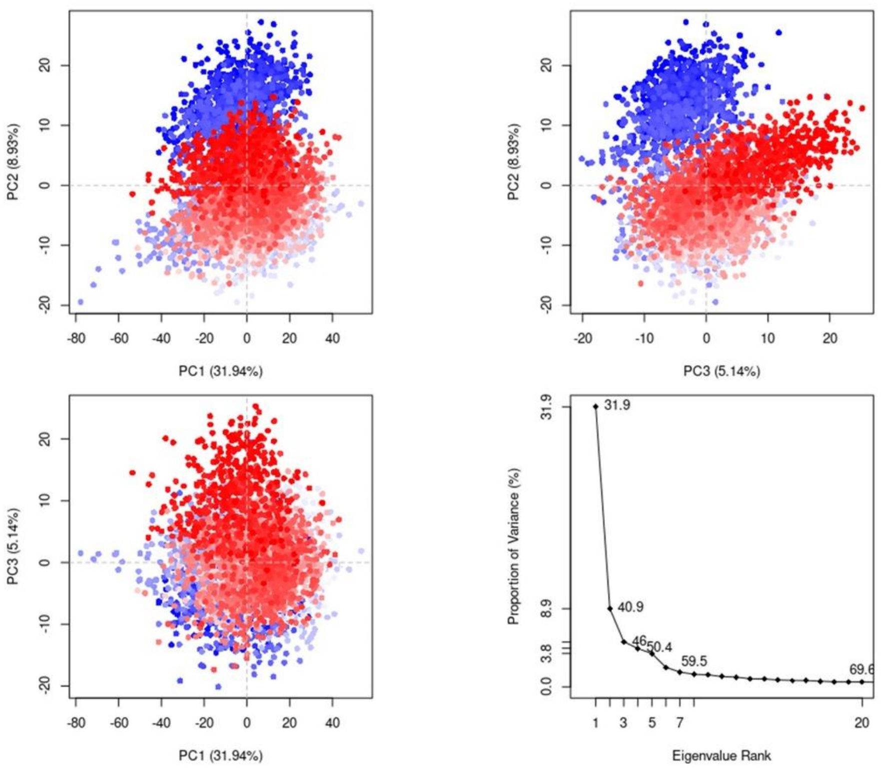

Figure S7- Principal component analysis for the generated for the reference molecule with docked MERS-protease protein target.
